# Association between multimorbidity patterns and activities of daily living function among older adults in China: a longitudinal population-based cohort study

**DOI:** 10.1186/s12877-025-06567-4

**Published:** 2025-11-10

**Authors:** Rui Li, Bizhong Che, Wenjuan Xiao, Jing Shu, Xiaowei Ma, Yanan Wang, Peng Nie, Youfa Wang, Xiaomin Sun

**Affiliations:** 1 Global Health Institute, School of Public Health, Xi’an Jiaotong University Health Science Center, Xi’an, 710061 China; 2 Xi’an Jiaotong University International Obesity and Metabolic Disease Research Center, Xi’an, 710061 China; 3 Department of Endocrinology, The First Affiliated Hospital of Xi’an Jiaotong University, Xi’an, Shaanxi Province China; 4 Mianyang City Center for Disease Control and Prevention, Institute of Chronic Disease Prevention and Control, Mianyang, 621000 China; 5 Med-X Institute, Center for Immunological and Metabolic Diseases, the First Affiliated Hospital of Xi’an Jiaotong University, Xi’an, 710061 China; 6 School of Economics and Finance, Xi’an Jiaotong University, Xi’an, 710061 China

**Keywords:** Older adults, Multimorbidity pattern, Activities of daily living, Socioeconomic disparity

## Abstract

**Background:**

Older individuals are vulnerable to multiple non-communicable chronic diseases, leading to an increased risk of a decline in activities of daily living (ADLs) disability. Whether this association is affected by sociodemographic factors remains unclear. This study aimed to identify multimorbidity patterns and explore their association with ADLs.

**Methods:**

This study included 14,018 older adults (≥ 60 years) from five waves of the China Health and Retirement Longitudinal Study (2011–2020). Multimorbidity patterns were identified among 13 non-communicable chronic diseases using exploratory factor analysis. Associations between multimorbidity patterns and ADLs (basic ADL, BADL; instrumental ADL, IADL) were examined using mixed-effects models. Stratified and interaction analyses were used to explore the influence of sociodemographic factors on these associations.

**Results:**

The prevalence of IADL disability among older individuals in China increased from 2011 to 2020.The prevalence of multimorbidity increased steadily from 2011 to 2018 but decreased in 2020. Four multimorbidity patterns were identified: visceral-skeletal, respiratory system, neurodegenerative, and cardiometabolic diseases. Higher factor scores of multimorbidity patterns were associated with increased risk of BADL and IADL disability, particularly for neurodegenerative diseases pattern (T3 vs. T1, BADL: OR 1.44, 95% CI 1.23–1.70; IADL: OR 1.66, 95% CI 1.42–1.94). The positive associations between neurodegenerative diseases and BADL and IADL disability were stronger among urban residents. The inverse associations between cardiometabolic diseases and IADL disability were stronger among educated than illiterate individuals.

**Conclusion:**

Multimorbidity was prevalent and independently associated with the risk of a decline in basic and instrumental activities of daily living among older individuals in China. Residential area and education level modified these associations.

**Trial registration:**

This study was approved by the Biomedical Ethics Committee of Peking University (NO. IRB00001052-11015).

**Supplementary Information:**

The online version contains supplementary material available at 10.1186/s12877-025-06567-4.

## Key points

• The prevalence of IADL disability among older individuals in China increased from 2011 to 2020.

• The prevalence of multimorbidity among older individuals in China increased from 2011 to 2018 but decreased in 2020.

• Multimorbidity was prevalent and independently associated with the risk of a decline in basic and instrumental ADL.

• This association was affected by residential area (urban vs. rural) and education level.

**Supplementary Information:**

The online version contains supplementary material available at 10.1186/s12877-025-06567-4.

## Introduction

Rapid aging of the population poses a serious global challenge. The United Nations reported an increase in the global population aged 65 years or older from 6.0% in 1990 to 9.3% in 2020 [1]. This trend was particularly pronounced in China. According to the 2021 census, individuals aged 65 years or older comprised 14.9% of the population [2, 3] and this number was projected to reach 30.0% (approximately 400 million people) by 2035 [4].

Older individuals are vulnerable to multiple non-communicable chronic diseases (NCDs), leading to an increased risk of a decline in activities of daily living (ADLs), mainly due to age-related physiological changes [5].The latest meta-analysis showed a 26% prevalence of disability among older adults in China, based on studies published between 1979 and 2021 [6]. Moreover, among older adults with cognitive impairment and/or ADL limitations, the number of individuals only experiencing ADL disability is projected to increase from 3.7 million in 2015 to 23.9 million in 2060, with an estimated annual growth rate of 12.2% [7]. Previous studies reported that increased functional disability were strongly associated with chronic diseases incidence (such as diabetes, stroke and heart disease) among older adults [8]. However, compared with single chronic conditions, multiple concurrent conditions pose greater challenges, including more complex healthcare needs, greater burden on healthcare systems, reduced quality of life [9], and elevated risks of disability [10] and mortality [11]. The prevalence of multimorbidity among individuals aged 60 years or older in high-income countries varies, ranging from 45.0% to 95.0% [12–16]. Similar trends have been observed in China, with multimorbidity rates among older individuals ranging from 40.0% to 82.0% [17–19]. However, previous studies were mainly conducted among community-dwelling older individuals or focused on a single time point.

Multimorbidity patterns are recognized as the concurrent presence of multiple diseases in an individual or group, with a certain degree of interrelation among these diseases [20]. A longitudinal study in Australia revealed that specific multimorbidity patterns, such as cardiovascular, cerebrovascular, and neurodegenerative disease patterns, influenced both basic and instrumental ADLs (BADL/IADL) among women aged 76–81 years [21]. Zhao et al. examined data from three rounds of the China Health and Retirement Longitudinal Study (CHARLS) conducted in 2011, 2013, and 2015 and reported that multimorbidity was associated with an increased risk of a decline in ADL among adults aged 45 years or older. However, this study defined the prevalence of multimorbidity as the presence of two or more chronic diseases, without considering the impact of the relationships among them [22]. Furthermore, although socioeconomic factors, such as education level, residential region, and marital status, are considered critical indices for healthcare and health status [23, 24], their impact on these associations has not been thoroughly evaluated.

Therefore, this study aimed to (1) examine the prevalence of multimorbidity and disability among older adults, (2) identify associations between multimorbidity patterns and ADLs, and (3) explore whether these associations were affected by sociodemographic factors.

## Materials and methods

### Study population and study design

We used data from the baseline and follow-up surveys of the CHARLS conducted in 2011, 2013, 2015, 2018, and 2020. CHARLS is a nationally representative survey of a cohort of Chinese people (aged ≥ 45 years) from 150 counties or districts and 450 villages or urban communities across 28 provinces. Participants were selected using multistage stratified probability proportionate to size sampling. A total of 18,229 respondents were enrolled at baseline in 2011. From 2013 to 2020, the number of newly added follow-up subjects was 3,524, 3,678, 575, and 127 respectively, with a total enrollment of 26,133 respondents. We excluded individuals younger than 60 years, those who had missing dependency variables, and those with data from only one wave of the study. Participants with baseline disabilities were excluded from the analysis. For individuals who experienced more than three assessments during the follow-up, the time of first recorded onset of disability was defined as the censoring point. Finally, 6609 and 7430 participants were included in the analyses of BADL and IADL, respectively (Figure S1).

All participants or their legal representatives provided written informed consent to participate in both baseline and follow-up surveys prior to participation. This study was approved by the Biomedical Ethics Committee of Peking University (IRB00001052-11015).

### ADL measurement

ADLs was classified into two categories: BADL and IADL. BADL disability was defined as dependence or needing assistance in at least one of the six BADL tasks of dressing, bathing, feeding, transferring (getting into or out of bed), toileting, and continence control. IADL disability was described as dependence in at least one IADL task: doing housework, preparing meals, shopping, taking medication, managing money, and making a phone call [25]. For each ADL task, participants were asked to choose one from the following four responses: (1) No, I do not have any difficulty; (2) I have difficulty, but I can still do it; (3) Yes, I have difficulty and need help; (4) I cannot do it. In binary logistic regression analyses, participants entered into the BADL or IADL disability group if they reported having difficulty, needing help, or cannot (i.e., had a response of 2–4) conduct one of the 6 BADL or IADL items [26].

### Multimorbidity and multimorbidity patterns

The surveys assessed 14 chronic diseases: (1) cancer or malignant tumor, excluding minor skin cancers; (2) liver disease, excluding fatty liver, tumors, and cancer, (3) heart attack, coronary heart disease, angina, congestive heart failure, or other heart problems; (4) stroke; (5) kidney disease, except for tumor or cancer; (6) chronic lung diseases, such as chronic bronchitis, emphysema, excluding tumors and cancer; (7) stomach or other digestive disease, excluding tumor and cancer; (8) arthritis or rheumatism; (9) asthma; (10) memory-related disease, including Alzheimer’s disease, cerebral atrophy, Parkinson’s disease; (11) emotional, nervous, or psychiatric problems; (12) hypertension; (13) diabetes or high blood sugar; and (14) dyslipidemia.

Hypertension was defined as systolic blood pressure (SBP) ≥ 140 mmHg and/or diastolic blood pressure (DBP) ≥ 90 mmHg [27]. Diabetes or hyperglycemia was defined as fasting blood glucose ≥ 7 mmol/L or a glycosylated hemoglobin ratio ≥ 6.50% [28]. Dyslipidemia was defined as low-density lipoprotein ≥ 3.64 mmol/l and/or high-density lipoprotein < 0.91 mmol/L and/or total cholesterol ≥ 5.72 mmol/L and/or triglycerides ≥ 1.70 mmol/L [29].

Multimorbidity was defined as the presence of two or more of the 14 chronic diseases. To avoid spurious associations, cancer or malignant tumor were excluded due to its prevalence being ≤ 1.0%. Thus, 13 chronic diseases were used to identify multimorbidity patterns.

#### Other variables

Socioeconomic status was evaluated based on education (illiterate [without any formal education], elementary school or below, or secondary school or above), marital status (married/cohabiting or not married [divorced/separated/widowed/never married]), and residential status (urban or rural) [30]. Other demographic characteristics were gender (male or female), age group (60–74 years or ≥ 75 years), smoking status (never smoked, former smoker, or current smoker), drinking status (never drank, less than monthly drinking, or monthly drinking or more), sleep status (< 7, 7–8, or > 8 h), and body mass index (BMI; weight/height^2^, kg/m^2^).

#### Statistical analysis

The baseline characteristics of the participants in 2011 were summarized using descriptive statistics. Continuous variables were expressed as mean ± standard deviation, and categorical variables were represented as n (%). Independent sample *t*-tests were performed to compare the difference between continuous variables. χ^2^-test or the Kruskal–Wallis test was used to examine the differences between categorical variables. All tests were two-tailed, with significance level set as *P* < 0.05. Logistic regression models were employed to test for trends in disability status across tertiles of factor scores for each multimorbidity pattern.

Multimorbidity patterns were identified using an exploratory factor analysis (EFA) based on the tetrachoric correlation matrix from the 13 NCDs. Sample adequacy was assessed using the Kaiser–Meyer–Olkin test, considered acceptable if the index was 0.645 (≥ 0.60), and Bartlett’s spherical test, considered adequate if the *P* < 0.01 [31]. The number of factors identified was based on their interpretability, with an eigenvalue ≥ 1. Variables were defined as being associated with a factor if it had a factor loading > 0.4 (Table S1). The tertiles (T1, T2, and T3) presented in Tables 2 and 3 represent the scores of multimorbidity patterns in ascending order, derived from EFA. Higher scores for different multimorbidity patterns indicate that participants are more likely to develop the diseases associated with those patterns. Multimorbidity patterns were also explored using latent class analysis (LCA) and the optimal number was selected based on the Bayesian Information Criterion (BIC), Akaike Information Criterion (AIC), entropy, and clinical interpretability.

Mixed-effects models were used to analyze the longitudinal associations between multimorbidity patterns and ADLs with odds ratios (ORs) and 95% confidence intervals (CIs). Binary coding of the BADL and IADL variables was used as the dependent variable. The time factor was quantified by survey waves and included in the model as a continuous fixed effect variable. Four models were fitted. Model 1 was adjusted for the factor scores of the other multimorbidity patterns. Model 2 was further adjusted for age, gender, education level, marital status, and urban or rural status. Model 3 included additional adjustments for weight status. Model 4 included additional adjustments for smoking status, drinking status, sleep duration and physical activity. Stratified and interaction analyses were used to explore the influence of sociodemographic characteristics on the associations between multimorbidity patterns and ADLs. Stratified analyses were conducted in two models: the first model was adjusted for age, gender, education level, marital status, residence, and BMI. The second model included additional adjustments for smoking status, drinking status, sleep duration, and physical activity. In both analyses, the stratified variables themselves were not included as covariates in the adjustments.

A significance level of *P* < 0.008 was set for the interaction terms involving six stratification variables, while *P* < 0.005 was used for those involving ten stratification variables. All statistical analyses were conducted using Stata 17.0 (Stata Corp, College Station, TX, USA).

## Results

### Baseline characteristics of the study population in the 2011 survey

The characteristics of the 7680 participants in 2011 according to multimorbidity were presented in Table 1. Compared with participants without multimorbidity, those with multimorbidity were older, more likely to be female and urban residents, non-smoker or former smokers, non-drinker, and tended to have higher percentages of unmarried, shorter sleep duration, higher BMI, and lower physical activity levels. Furthermore, participants with multimorbidity exhibited significantly higher percentage of both BADL and IADL disability than those without multimorbidity.

**Table 1:** Baseline characteristics of individuals aged ≥ 60 years in 2011

Characteristics	Total (*N* = 7680)	Non-multiple diseases (*N* = 1699)	Multiple diseases (*N* = 5981)	*P*
Age (years, mean ± SD)	68.5 ± 7.1	68.1 ± 7.2	68.6 ± 7.0	**0.008**
Age group (years, %)				0.830
60–74	6124(79.74%)	1357 (79.9%)	4767 (79.7%)	
≥ 75	1556(20.26%)	342 (20.1%)	1214 (20.3%)	
Gender (%)				** < 0.001**
Male	3850(50.13%)	975 (57.4%)	2875 (48.1%)	
Female	3830(49.87%)	724 (42.6%)	3106 (51.9%)	
Residential area (%)				0.059
Urban	3054(39.77%)	642 (37.8%)	2412 (40.3%)	
Rural	4626(60.23%)	1057 (62.2%)	3569 (59.7%)	
Marital status (%)				0.390
Married/Cohabiting	5973(71.81%)	1332 (78.6%)	4641 (77.6%)	
Not married	1703(22.19%)	363 (21.4%)	1340 (22.4%)	
Education level (%)				0.770
Illiterate	2845(37.08%)	616 (36.3%)	2229 (37.3%)	
Elementary School or Below	3355(43.73%)	750 (44.2%)	2605 (43.6%)	
Secondary School or Above	1472(19.19%)	329 (19.5%)	1143 (19.1%)	
BADL disability (%)				** < 0.001**
Yes	1889(32.00%)	216 (21.1%)	1673 (34.3%)	
No	4014(68.00%)	803 (78.8%)	3211 (65.7%)	
IADL disability (%)				** < 0.001**
Yes	2187(29.52%)	270 (17.0%)	1917 (32.6%)	
No	5221(70.48%)	1315 (83.0%)	3906 (67.4%)	
Smoking status (%)				** < 0.001**
Never smoked	4409(59.61%)	907 (57.3%)	3502 (60.2%)	
Former smoker	844(11.41%)	131 (8.3%)	713 (12.3%)	
Current smoker	2143(28.98%)	544 (34.4%)	1599 (27.5%)	
Drinking status (%)				**0.022**
Never	4600(60.51%)	947 (57.6%)	3653 (61.3%)	
Drinking, < 1 time/month	595(7.83%)	132 (8.1%)	463 (7.8%)	
Drinking, ≥ 1 time/month	2407(31.66%)	564 (34.3%)	1843 (30.9%)	
Sleep Hours (%)				**0.014**
< 7	3074(53.82%)	635 (45.8%)	2439 (50.1%)	
7–8	2020(35.36%)	598 (43.1%)	1962 (40.3%)	
> 8	618(10.82%)	153 (11.1%)	465 (9.6%)	
BMI (kg/m^2^, mean ± SD)	22.8 ± 3.9	21.9 ± 3.5	23.2 ± 3.9	** < 0.001**
Physical activity (%)				
High	801(27.19%)	131 (21.0%)	670 (28.9%)	** < 0.001**
Middle	1493(50.68%)	318 (50.9%)	1175 (50.6%)	
Low	652(20.13%)	176 (28.1%)	476 (20.5%)	

*P* < 0.05 indicated in bold

**Fig. 1:**
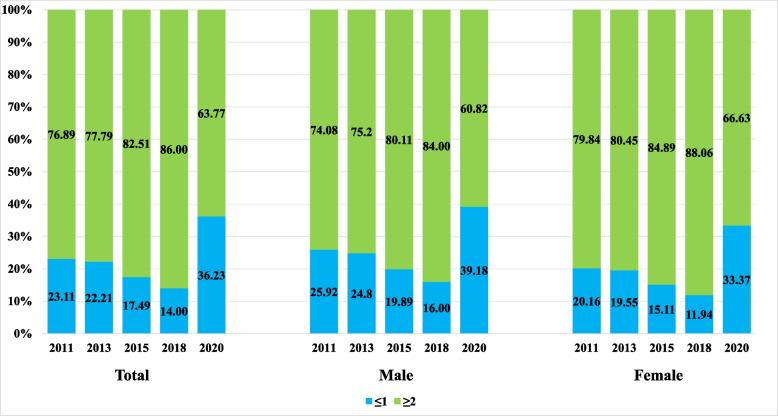
Distribution of chronic disease numbers by gender

Figure 1 illustrates the distribution of chronic diseases according to sex. The proportion of participants with two or more diseases increased from 76.9% in 2011 to 86.0% in 2018 but decreased to 63.4% in 2020. In each wave, women were more likely to have two or more diseases. The prevalence of ADL and BADL disability both increased from 2011 to 2015, declined slightly in 2018, and then rose again in 2020. In contrast, the prevalence of IADL disability steadily increased from 2011–2020. (Table S2).

### Extraction of multimorbidity patterns among older individuals

Four multimorbidity patterns were identified using EFA: visceral-skeletal diseases, including stomach or other digestive disorders, liver disorders, kidney disorders, and arthritis or rheumatism; respiratory diseases, including asthma and chronic lung disorders; neurodegenerative diseases, including emotional and psychiatric problems, memory-related disorders, and stroke; and cardiometabolic diseases, including hypertension, dyslipidemia, heart disease, and stroke (Table S3).

### Longitudinal associations between multimorbidity patterns and ADL

Table 2 presents the association between BADL disability and multimorbidity patterns. After adjusting for the other 3 multimorbidity patterns, BADL disability was positively associated with visceral-skeletal, respiratory system, and neurodegenerative diseases. These associations remained significant when additional adjustment was made for sociodemographic factors. In Model 4 that further adjusted for BMI, smoking status, drinking status, sleep duration and physical activity, ORs (95% CIs) for the highest tertiles of factor score of visceral skeletal diseases, respiratory system diseases, and neurodegenerative diseases were 1.17 (1.0–1.37), 1.33 (1.15–1.54), and 1.44 (1.23–1.70), respectively, for BADL disability, compared with the lowest tertiles (all P_trend_ < 0.01). Each increase in the factor scores of visceral-skeletal, respiratory systems, and neurodegenerative diseases was associated with a 22% (OR 1.22, 95% CI 1.03–1.45), 43% (OR 1.43, 95% CI 1.17–1.74), and 167% (OR 2.67, 95% CI 1.89–3.77) higher risk of BADL disability, respectively. BADL disability was inversely associated with cardiometabolic diseases. Each increase in the factor score of cardiometabolic diseases was associated with a 35% (OR 0.65 95% CI 0.55–0.76) lower risk of BADL disability.

**Table 2:** Longitudinal associations between multimorbidity patterns and BADL disability based on CHARLS 2011–2020 (*N* = 6609)

	**Multimorbidity Patterns (OR [95% CI])**			
	**Visceral-Skeletal Diseases**	**Respiratory System Diseases**	**Neurodegenerative Diseases**	**Cardiometabolic Diseases**
Model 1				
Factor Score				
T1	1.00	1.00	1.00	1.00
T2	1.04 (0.94–1.14)	**1.37 (1.25–1.51)**	0.95 (0.86–1.05)	1.01 (0.91–1.10)
T3	**1.22 (1.10–1.35)**	**1.44 (1.32–1.59)**	**1.46 (1.32–1.61)**	1.01 (0.91–1.11**)**
Time	**3.54(3.41–3.67)**			
*P*_trend_	** < 0.001**	** < 0.001**	** < 0.001**	0.969
Per Increase in Factor Score	**1.24 (1.12–1.38)**	**1.59 (1.41–1.79)**	**2.87 (2.32–3.54)**	**0.88 (0.81–0.97)**
Time	**3.54(3.41–3.67)**			
Model 2				
Factor Score				
T1	1.00	1.00	1.00	1.00
T2	1.06 (0.95–1.17)	**1.30 (1.18–1.43)**	0.98 (0.88–1.08)	0.90 (0.82–1.01)
T3	**1.30 (1.17–1.44)**	**1.31 (1.19–1.45)**	**1.47 (1.33–1.63)**	0.94 (0.84–1.04)
Time	**3.63 (3.49–3.78)**			
*P*_trend_	** < 0.001**	** < 0.001**	** < 0.001**	0.224
Per Increase in Factor Score	**1.37 (1.23–1.53)**	**1.39 (1.23–1.58)**	**2.80 (2.25–3.48)**	**0.84 (0.76–0.93)**
Time	**3.66(3.51–3.80)**			
Model 3				
Factor Score				
T1	1.00	1.00	1.00	1.00
T2	1.08 (0.96–1.22)	**1.31 (1.17–1.47)**	0.94(0.84–1.06)	**0.87 (0.78–0.98)**
T3	**1.38 (1.23–1.55)**	**1.32 (1.18–1.46)**	**1.39 (1.24–1.57)**	**0.86 (0.76–0/98)**
Time	**3.61(3.45–3.77)**			
*P*_trend_	** < 0.001**	** < 0.001**	** < 0.001**	**0.017**
Per Increase in Factor Score	**1.47 (1.29–1.66)**	**1.42 (1.23–1.64)**	**2.54 (1.98–3.27)**	**0.79 (0.70–0.88)**
Time	**3.62(3.46–3.78)**			
Model 4				
Factor Score				
T1	1.00	1.00	1.00	1.00
T2	1.03(0.88–1.20)	**1.36(1.17–1.59)**	0.97(0.83–1.14)	**0.76(0.65–0.89)**
T3	**1.17(1.01–1.37)**	**1.33(1.15–1.54)**	**1.44(1.23–1.70)**	**0.72(0.61–0.85)**
Time	**6.37(5.82–6.96)**			
*P*_trend_	**0.019**	**0.002**	** < 0.001**	** < 0.001**
Per Increase in Factor Score	**1.22(1.03–1.45)**	**1.43(1.17–1.74)**	**2.67(1.89–3.77)**	**0.65(0.55–0.76)**
Time	**6.41(5.86–7.01)**			

*P* < 0.05 indicated in bold

Model 1: adjusted for the score of other three multimorbidity patterns

Model 2: in addition to Model 1, adjusted for age, gender, education level, marital status, and urban or rural residence

Model 3: in addition to Model 2, adjusted for BMI

Model 4: in addition to Model 3, adjusted for smoking status, drinking status, sleep duration and physical activity

The associations between IADL disability and multimorbidity patterns are presented in Table 3. The multivariable-adjusted ORs (95% CIs) for the highest tertiles of factor score of visceral-skeletal, respiratory system, neurodegenerative, and cardiometabolic diseases for IADL disability were 1.18 (1.02–1.37), 1.68 (1.46–1.94), 1.66 (1.42–1.94), and 0.86 (0.74–1.02), respectively, for IADL disability, compared with the lowest tertiles (all P_trend_ < 0.01). Each increase in the factor scores of respiratory system, and neurodegenerative diseases was associated with a 103% (OR 2.03, 95% CI 1.65–2.49), and 409% (OR 5.09, 95% CI 3.53–7.34) higher risk of IADL disability, respectively. But each increase in the factor scores of cardiometabolic diseases was associated with a 28% (OR 0.72 95% CI 0.62–0.83) lower risk of IADL disability.

**Table 3:** Longitudinal associations between multimorbidity patterns and IADL disability based on CHARLS 2011–2020 (*N* = 7430)

	**Multimorbidity Patterns (OR [95% CI])**							
	**Visceral-Skeletal Diseases**			**Respiratory System Diseases**	**Neurodegenerative Diseases**		**Cardiometabolic Diseases**	
Model 1								
Factor Score								
T1	1.00			1.00	1.00		1.00	
T2	**1.20 (1.10–1.30)**			**1.46 (1.34–1.59)**	**0.96 (0.88–1.05)**		**1.08 (0.99–1.17)**	
T3	**1.20 (1.11–1.31)**			**1.73 (1.59–1.88)**	**1.42 (1.29–1.55)**		**1.07 (0.97–1.17)**	
Time	**2.85 (2.77–2.94)**							
* P*_trend_	** < 0.001**			** < 0.001**	** < 0.001**		0.606	
Per Increase in Factor Score	**1.18 (1.07–1.29)**			**2.20 (1.96–2.48)**	**3.77 (3.05–4.68)**		0.93 (0.85–1.01)	
Time	**2.86 (2.77–2.95)**							
Model 2								
Factor Score								
T1	1.00			1.00	1.00		1.00	
T2	**1.12 (1.06–1.19)**			**1.21 (1.14–1.28)**	0.97 (0.91–1.03)		1.05(0.99–1.11)	
T3	**1.29 (1.22–1.37)**			**1.26 (1.19–1.33)**	**1.38 (1.29–1.46)**		**1.13 (1.06–1.20)**	
Time	**1.38 (1.36–1.40)**							
* P*_trend_	** < 0.001**			** < 0.001**	** < 0.001**		**0.020**	
Per Increase in Factor Score	**1.20 (1.08–1.33)**			**1.95 (1.72–2.20)**	**4.05 (3.23–5.08)**		**0.82 (0.75–0.90)**	
Time	**3.31 (3.19–3.43)**							
Model 3								
Factor Score								
T1	1.00			1.00	1.00		1.00	
T2	**1.16 (1.08–1.24)**			**1.23(1.15–1.31)**	0.97 (0.91–1.04)		1.04 (0.98–1.12)	
T3	**1.34 (1.25–1.44)**			**1.26 (1.18–1.34)**	**1.26 (1.17–1.35)**		**1.12 (1.04–1.20)**	
Time	**1.36 (1.34–1.39)**							
* P*_trend_	** < 0.001**			** < 0.001**	** < 0.001**		0.169	
Per Increase in Factor Score	**1.26 (1.12–1.42)**			**1.84 (1.60–2.12)**	**3.28 (2.53–4.25)**		**0.86 (0.77–0.95)**	
Time	**3.17 (3.05–3.30)**							
Model 4								
Factor Score								
T1		1.00	1.00			1.00		1.00
T2		**1.19(1.03–1.38)**	**1.68(1.46–1.93)**			1.13(0.98–1.32)		0.87(0.75–1.01)
T3		**1.18(1.02–1.37)**	**1.68(1.46–1.94)**			**1.66(1.42–1.94)**		0.86(0.74–1.02)
Time		**5.25(4.55–5.51)**						
P_trend_		**0.040**	** < 0.001**			** < 0.001**		**0.006**
Per Increase in Factor Score		1.03(0.87–1.21)	**2.03(1.65–2.49)**			**5.09(3.53–7.34)**		**0.72(0.62–0.83)**
Time		**5.07(4.70–5.46)**						

*P* < 0.05 indicated in bold

Model 1: adjusted for the score of other three multimorbidity patterns

Model 2: in addition to Model 1, adjusted for age, gender, education level, marital status, and urban or rural residence

Model 3: in addition to Model 2, adjusted for BMI

Model 4: in addition to Model 3, adjusted for smoking status, drinking status, sleep duration and physical activity

The LCA finally identified a 3-class model, consisting of respiratory, metabolic, and musculoskeletal-organ disease patterns (Table S5 and S6). When liver disease, emotional/mental disorders, and memory-related diseases were excluded due to their low prevalence, a similar 3-class model were still obtained (Table S6). Moreover, the associations between these LCA-derived disease patterns and ADL were consistent with those observed in the patterns derived from EFA (Tables S7 and S8).

### Stratified analysis

**Fig. 2:**
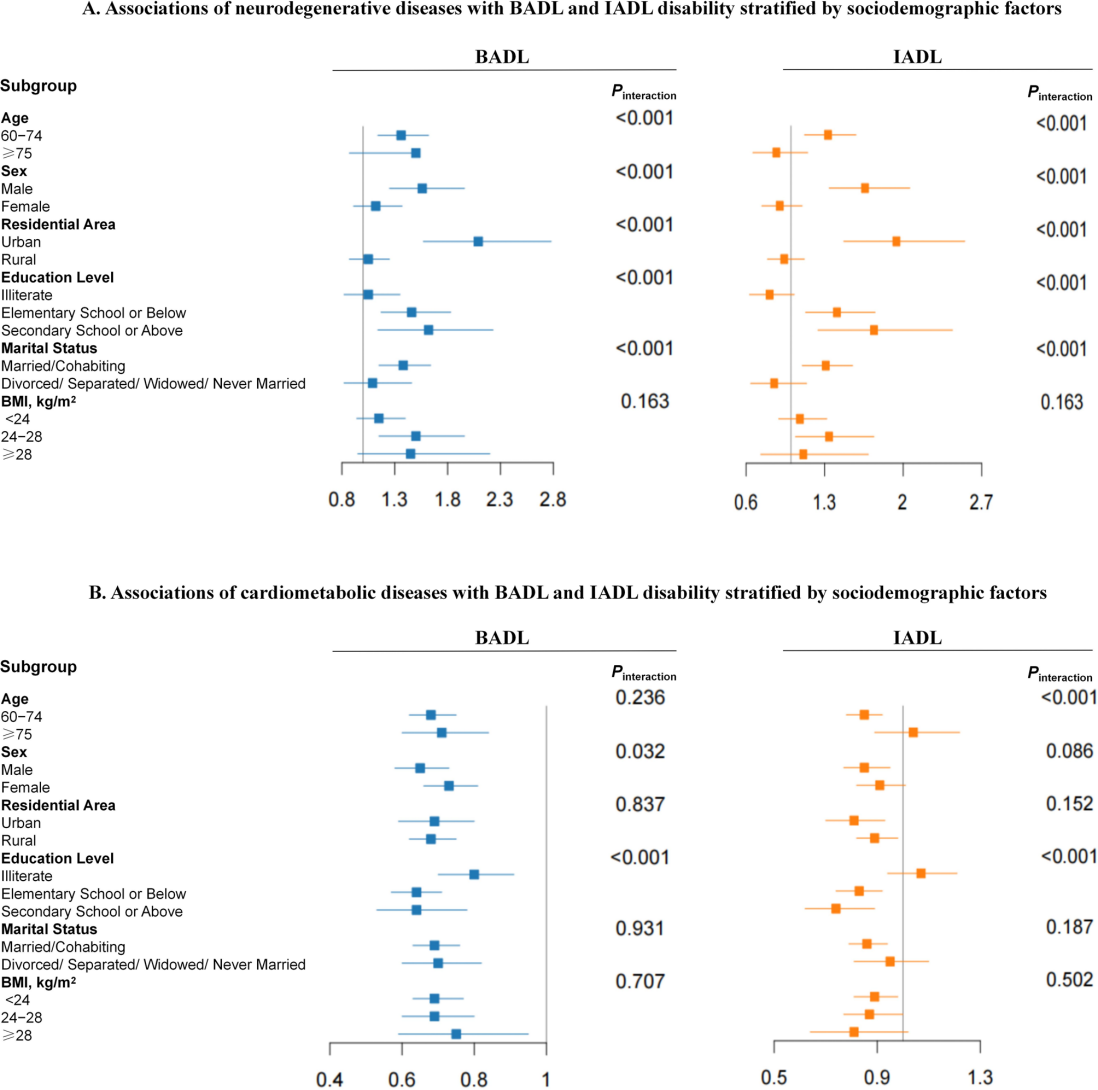
Associations of neurodegenerative diseases pattern (**A**) and cardiometabolic diseases pattern (**B**) with BADL and IADL disability stratified by sociodemographic factors

We conducted a stratified analysis of the associations of the two most significant multimorbidity patterns (neurodegenerative and cardiometabolic diseases) with BADL and IADL disability according to potential risk factors. As shown in Fig. 2A, the positive associations between BADL and IADL disability and neurodegenerative diseases were stronger among urban individuals (both *P*_interaction_ < 0.001). The neurodegenerative disease pattern was associated with a significantly higher risk of IADL disability among participants aged 60–74 years, educated participants, and married or partnered participants (all *P*_interaction_ ≤ 0.001). The inverse associations between cardiometabolic diseases and BADL and IADL disability were stronger among educated individuals (both *P*_interaction_ < 0.001). The cardiometabolic disease pattern was associated with a significantly lower risk of IADL disability among participants aged 60–74 years (*P*_interaction_ < 0.001; Fig. 2B). After adjusting for BMI, smoking status, drinking status, sleep duration and physical activity, the inverse associations between cardiometabolic diseases and IADL disability were stronger among educated individuals (*P*_interaction_ = 0.003; Figure S2). No significant interaction was observed between disability and these risk factors in the other multimorbidity patterns. (*P*_interaction_ > 0.005).

## Discussion

This study used nationally representative data from the 2011–2020 CHARLS to identify the patterns of chronic multimorbidity and explore its associations with ADLs prevalence among adults aged 60 years or older in China. Overall, the prevalence of ADL and BADL disability increased from 2011 to 2020, with a slight decline observed in 2018. While we observed a steady increase in the prevalence of IADL disability from 2011 to 2020. Notably, the rates of ADL disability were significantly higher in 2020 than other years, probably due to the severe impact of COVID-19 on ADLs. Previous studies reported that COVID-19 survivors had a higher risk of impaired ADLs [32, 33]. The proportion of participants with two or more diseases increased annually from 2011 to 2018 but decreased in 2020. This may be the result of deaths among older adults with multimorbidity occurring in 2020, which was the period when the world was affected by the COVID-19 pandemic. A previous study revealed a significantly escalated risk of poor prognosis among patients infected with COVID-19 who had two or more comorbidities compared with those who had no or a single comorbidity [34]. Furthermore, most comorbidities were associated with a higher risk of death due to COVID-19 [35]. Moreover, older patients and those with multiple chronic diseases had poor prognosis for COVID-19 and were prone to death [36].

Previous studies have found that people carrying ≥ 4 diseases had an increased risk of developing ADL disability compare to those without chronic diseases [37]. The dose–response associations between the number of diseases and the degree of future disability means that the more chronic diseases accumulate, the greater the risk of functional limitations [38].However, the disease profile of older people is complex, with different pathogenic mechanisms for different diseases. The extraction of multimorbidity patterns facilitates the identification of diseases with the same etiology and further explores the relationship between disability and different multimorbidity patterns. This study identified four primary multimorbidity patterns: visceral-skeletal, respiratory, neurodegenerative, and cardiometabolic. Each pattern was independently associated with the risk of BADL and IADL disability. This was in line with previous studies using factor analysis to identify natural groups of diseases among older adults, which identified three to five patterns [39–41]. The neurodegenerative disease pattern mainly comprised stroke and cognitive psychiatric disorders, which posed the highest risk for both BADL and IADL disabilities. Prior studies comparing multiple chronic conditions have shown that stroke was the most disabling disease [42, 43]. Stroke onset has a serious impact on a patient’s mobility and memory, preventing the patient from performing ADL [44]. Previous studies have shown a positive association between cardiometabolic diseases and ADL disability risk [18, 43]; however, our results suggested that cardiovascular disease was associated with low BADL disability. This inconsistency may be because previous studies did not use the most current data or relied solely on cross-sectional data.

In addition, this study included 2020 survey data, encompassing a period during the COVID-19 pandemic. Thus, surviving older adults may face a higher risk of impaired ADLs due to the effects of COVID-19, potentially introducing survivor bias. To assess the survival bias of the association of cardiometabolic disease with BADL and IADL, we conducted a sensitivity analysis which was excluded the 2020 survey from the analysis. The analysis found that high scores of cardiovascular diseases pattern was more likely to increase the risk of disability in BADL, consistently with previous evidence [18, 21] (Table S9), while no substantial changes were observed in the associations with IADL (Table S10). Multiple studies have confirmed that COVID-19 infection, even mild cases, can have persistent impacts on the physical function of older adults and increase the risk of disability. On the other hand, during the pandemic, patients with hypertension, heart disease, and diabetes were regarded as high-risk groups and thus gained access to more medical resources and early intervention opportunities, which may have improved disease control and indirectly reduced the risk of disability in such patients [45, 46]. Additionally, the heightened perception of health risks brought about by COVID-19 significantly enhanced treatment adherence among some patients (e.g., those with cardiovascular diseases) [47]. In contrast, individuals with respiratory or neurological diseases may experience more persistent functional impairments due to the complexity of their treatment regimens and lower rehabilitation adherence [48, 49]. These aforementioned factors may have contributed to the improvement of cardiometabolic multimorbidity, thereby reducing the prevalence of complications associated with such diseases and leading to the reversed results observed when the 2020 data were included in the analysis.

Stratified analyses showed that the association between cardiometabolic disease pattern and BADL disability were only existed among individuals with lower levels of education. Socioeconomic status is a broad concept that mainly includes education, income, and occupation, which was found to be linked with health [50]. Many studies have shown that poor socioeconomic status (lower levels of income and education, higher rates of poverty and unemployment) were more likely to increased risk of ADL disability [51, 52].

The inverse associations between cardiometabolic diseases and BADL and IADL disability were stronger among educated than illiterate individuals. This is in line with the results of previous studies [53, 54]. This may be because educated people are more likely to receive health education and practice a healthy lifestyle, such as avoiding smoking and drinking and adhering to a healthy diet, which in turn reduces the negative consequences of the disease and the risk of disability [55, 56]. Furthermore, this study found that the positive associations between neurodegenerative diseases and BADL and IADL disability were stronger among urban than rural residents. Previous studies have shown that urban residents typically have access to sufficient health resources, advanced healthcare services, and abundant opportunities for rehabilitation and physical activity [57], thereby reducing the risk of disability. In contrast, older adults living in rural areas generally have limited medical resources and opportunities for rehabilitation. Coupled with the physical demands of agricultural work, these conditions exacerbate the risk of disability. After adjusting for s BMI, smoking status, drinking status, sleep duration and physical activity, the inverse associations between cardiometabolic diseases and IADL disability remained stronger only among individuals with higher education. One possible explanation is that lifestyle factors such as smoking and alcohol consumption exert a stronger influence on cardiometabolic conditions [58, 59]. Individuals with higher education levels may be more likely to adopt healthier lifestyles and manage these risk factors more effectively, thereby mitigating IADL impairment in the presence of cardiometabolic diseases [60].

This study employed mixed-effects models to examine the associations between multimorbidity patterns and various outcome variables, such as incapacitation. This approach considered individual heterogeneity and the characteristics of repeated-measures data, enabling comprehensive data use. Unlike previous studies, which focused primarily on the number of chronic multimorbidities, our analysis explored the differential impacts of various disease combinations on outcome variables. An important aspect of this study is the incorporation of the results of the 2020 updated data, which reflect the current multimorbidity situation in China.

Our study had some limitations. First, disease status was mainly self-reported and the range of chronic diseases available in CHARLS was limited, which may have introduced reporting bias and constrained a more comprehensive analysis of multimorbidity patterns. Second, due to limitations in the original data, behavioral variables were simplified, potentially limiting the control of lifestyle-related confounding factors. Third, the follow-up period was relatively short, which might have affected the comprehensiveness of the findings. Fourth, since alcohol consumption was unavailable for all waves, we used drinking frequency as a proxy for alcohol consumption. Future studies should incorporate objective measurements, more detailed behavioral assessments, and longer follow-up periods to enhance the accuracy and validity of these variables.

Moreover, differences in the construction methods of multimorbidity patterns may affect the selection of disease variables, which could in turn limit the generalizability of the results. To address this, we applied both EFA and latent class analysis LCA, and identified three broadly similar disease patterns across the two approaches, suggesting a degree of consistency. However, EFA classifies diseases based on their co-occurrence patterns rather than clinical criteria, which may mask the heterogeneity in functional impacts between "memory-impairment-related diseases" and "emotional/mental disorders". Future studies should incorporate more detailed diagnostic data or samples from specific high-risk populations. This study did not capture dynamic changes in functional states, which could be explored in future research using multi-state models.Since the follow-up period of our study overlapped with the COVID-19 pandemic, pandemic-related disruptions may have influenced the results, and the observed association between cardiovascular disease and ADL disability should therefore be interpreted with caution.Finally, the participants included in our study were more likely to be female, married, from rural areas, non-smokers, and to have higher levels of education and physical activity. Thus, the findings may not be generalizable to the entire older populations, which might introduce selection bias. Future studies can optimize this by expanding the sampling framework and including special samples of institutionalized older adults and older adults. Meanwhile, it is suggested that other older adults’ health cohorts should pay attention to the systematic inclusion of such special subpopulations to more comprehensively reflect health disparities among the older population.

As China’s demographic landscape evolves, with longer life expectancies and lower birth rates, addressing the challenges of an aging population has become increasingly critical. This study underscores the need for an in-depth exploration of the natural progression of multimorbidity patterns. The results of this study could guide future strategies for promoting healthy aging, personalized healthcare, and effective community management to maintain the functional abilities of older adults. Thus, adapting multimorbidity prevention and control strategies used in high-income countries to China’s unique context is essential. This approach could help manage the challenges posed by an aging population and foster healthy and fulfilling aging. Furthermore, the older population’s knowledge of disease risk and ability to implement healthy lifestyles should be increased to mitigate disempowerment caused by educational disparities and improve the overall health and well-being of the aging population.

## Conclusions

This study demonstrated that multimorbidity patterns were independently associated with the risk of BADL and IADL disability among older individuals in China. Residential area and education level affected these associations.

## Supplementary Information

Supplementary Material 1

## Data Availability

The datasets generated and/or analyzed during the current study are not publicly available due belonging to a team data but are available from the corresponding author on reasonable request. The official website for data access is: http://charls.pku.edu.cn/.

## Abbreviations

NCDs: Non-communicable chronic diseases
ADLs: Activities of daily living
BADL: Basic activities of daily living
IADL: Instrumental activities of daily living
EFA: Exploratory factor analysis
LCA: Iatent class analysis
BIC: Bayesian Information Criterion
AIC: Akaike Information Criterion
