# Supplementary material for: Association between multimorbidity patterns and activities of daily living function among older adults in China: a longitudinal population-based cohort study
Source: BMC Geriatr. 2025 Nov 10;25:885. doi: 10.1186/s12877-025-06567-4 (PMC12604309; doi:10.1186/s12877-025-06567-4)
Supplement: Supplementary file 1 — Supplementary Material 1 [file 12877_2025_6567_MOESM1_ESM.docx]

**Supplemental materials**

**Table S1.** Factor analysis of eigenvalues and cumulative variance contribution rates before and after chronic disease imputation (sensitivity analysis)

| Factor | Before Imputation (N=27,685) | | After Imputation (N=45,585) | |
| --- | --- | --- | --- | --- |
|  | Eigenvalues | Cumulative Variance Contribution Rates | Eigenvalues | Cumulative Variance Contribution Rates |
| Factor 1 | **3.315** | 0.255 | **3.151** | 0.241 |
| Factor 2 | **1.632** | 0.381 | **1.604** | 0.365 |
| Factor 3 | **1.217** | 0.474 | **1.252** | 0.462 |
| Factor 4 | **1.147** | **0.562** | **1.182** | **0.553** |
| Factor 5 | 0.917 | 0.633 | 0.991 | 0.629 |
| Factor 6 | 0.841 | 0.697 | 0.862 | 0.695 |
| Factor 7 | 0.787 | 0.758 | 0.770 | 0.754 |
| Factor 8 | 0.686 | 0.811 | 0.687 | 0.807 |
| Factor 9 | 0.632 | 0.859 | 0.654 | 0.858 |
| Factor 10 | 0.572 | 0.903 | 0.571 | 0.901 |
| Factor 11 | 0.544 | 0.945 | 0.553 | 0.944 |
| Factor 12 | 0.467 | 0.981 | 0.482 | 0.981 |
| Factor 13 | 0.237 | 1.000 | 0.240 | 1.000 |

Eigenvalues >1 and cumulative variance contribution >50% indicated in bold.

| **Table S2.** Prevalence of disability in the older adults from 2011-2020 | | | | | | | | | |
| --- | --- | --- | --- | --- | --- | --- | --- | --- | --- |
| Year | ADLs Disability | | | BADL Disability | | | IADL Disability | | |
|  | Yes | NO | prevalence | Yes | NO | prevalence | Yes | NO | prevalence |
| 2011(N=7680) | 2779 | 4683 | 36.18% | 1889 | 4014 | 24.59% | 2187 | 5275 | 28.47% |
| 2013(N=8733) | 3786 | 4552 | 43.35% | 2099 | 4654 | 25.05% | 3313 | 5025 | 37.94% |
| 2015(N=9623) | 4318 | 4902 | 44.87% | 2628 | 4701 | 27.31% | 3684 | 5536 | 38.28% |
| 2018(N=9634) | 4317 | 4996 | 44.81% | 2555 | 5093 | 26.52% | 3775 | 5538 | 39.18% |
| 2020(N=9914) | 7878 | 2032 | 79.46% | 6944 | 2966 | 70.04% | 6551 | 3359 | 66.07% |

**Table S3.** Factor loadings of 13 chronic diseases on four multimorbidity patterns

| Chronic Diseases | Factor Loadings | | | |
| --- | --- | --- | --- | --- |
|  | Visceral-Skeletal Diseases | Respiratory System Diseases | Neurodegenerative Diseases | Cardiometabolic Diseases |
| Hypertension | -0.078 | 0.027 | -0.022 | **0.813** |
| Dyslipidemia | 0.241 | -0.120 | -0.035 | **0.576** |
| Diabetes mellitus or elevated blood glucose | -0.110 | 0.085 | -0.071 | **0.430** |
| Chronic lung disorders | 0.097 | **0.903** | -0.030 | -0.018 |
| Liver disorders | **0.629** | 0.003 | 0.028 | 0.088 |
| Heart disease | 0.310 | 0.215 | 0.131 | **0.407** |
| Stroke | -0.053 | -0.076 | **0.538** | **0.461** |
| Kidney disorders | **0.600** | 0.025 | 0.128 | 0.108 |
| Stomach or other digestive disorders | **0.745** | 0.001 | 0.029 | -0.170 |
| Emotional and psychiatric problems | 0.096 | 0.030 | **0.796** | -0.191 |
| Memory-related disorders | -0.030 | 0.043 | **0.794** | 0.142 |
| Arthritis or rheumatism | **0.636** | 0.061 | -0.111 | 0.027 |
| Asthma | -0.051 | **0.932** | 0.053 | 0.0001 |

Kaiser–Meyer–Olkin value=0.645; Bartlett’s test of sphericity, *P*<0.001. Rotation of the factor axes was performed using the promax method.

Factor loadings in exploratory factor analysis indicate the strength of association between each variable (disease) and each factor (disease pattern), with absolute values of factor loadings greater than 0.4 indicating greater strength of association (indicated in bold).

| **Table S4.** Comparison of fit indices for selecting optimal number of classes in Latent Class Analysis models | | | |
| --- | --- | --- | --- |
| LCA class | AIC | BIC | Standardized Entropy Value |
| 3 class | 999420.43 | 999818.34 | 0.40 |
| 4 class | 994751.10 | 995281.64 | 0.35 |
| 5 class | 991290.20 | 991953.37 | 0.39 |
| AIC: Akaike Information Criterion; BIC: Bayesian Information Criterion; LCA: Latent Class Analysis. | | | |

| **Table S5.** Classification of 13 chronic disease multimorbidity patterns identified by Latent Class Analysis | | | | | |
| --- | --- | --- | --- | --- | --- |
|  | **Class 1** | **Class 2** | **Class 3** | **Class 4** | **Over all** |
| multimorbidity patterns | Relatively healthy | Visceral-Skeletal Diseases | Metabolic Diseases | Respiratory System Diseases |  |
| Prevalence within each class (%) | | | | | |
| Hypertension | 21.94 | 56.84 | **99.56** | 56.77 | 44.38 |
| Dyslipidemia | 24.38 | 60.25 | **82.87** | 50.19 | 42.8 |
| Diabetes mellitus or elevated blood glucose | 31.08 | 42.06 | **45.2** | 41.17 | 36.07 |
| Chronic lung disorders | 5.64 | 31.77 | 8.22 | **98.59** | 14.33 |
| Liver disorders | 2.74 | 21.94 | 3.95 | 12.47 | 6.48 |
| Heart disease | 3.71 | **46.77** | 31.05 | 40.56 | 17.60 |
| Stroke | 0.76 | 9.67 | 13.81 | 8.01 | 5.08 |
| Kidney disorders | 3.83 | **30.49** | 8.12 | 19.26 | 9.62 |
| Stomach or other digestive disorders | 21.12 | **84.24** | 7.87 | 43.60 | 29.59 |
| Emotional and psychiatric problems | 0.95 | 6.65 | 1.61 | 5.63 | 2.19 |
| Memory-related disorders | 0.69 | 8.65 | 6.61 | 10.31 | 3.55 |
| Arthritis or rheumatism | 27.73 | **85.98** | 35.69 | 58.98 | 39.99 |
| Asthma | 0.75 | 4.07 | 1.80 | **99.30** | 5.71 |
| The bolded parts refer to the diseases that have been identified. | | | | |  |

| **Table S6.** Classification of 9 chronic disease multimorbidity patterns identified by Latent Class Analysis | | | | | |
| --- | --- | --- | --- | --- | --- |
|  | **Class 1** | **Class 2** | **Class 3** | **Class 4** | **Over all** |
| multimorbidity patterns | Relatively healthy | Visceral-Skeletal Diseases | Metabolic Diseases | Respiratory System Diseases |  |
| Prevalence within each class (%) | | | | | |
| Hypertension | 18.92 | 54.54 | **99.96** | 63.38 | 44.38 |
| Dyslipidemia | 21.62 | 64.25 | **82.80** | 45.86 | 42.8 |
| Diabetes mellitus or elevated blood glucose | 31.55 | 35.45 | **46.29** | 44.55 | 36.07 |
| Chronic lung disorders | 5.01 | 27.26 | 5.94 | **92.61** | 14.33 |
| Heart disease | 4.04 | **38.41** | 29.20 | **47.58** | 17.60 |
| Kidney disorders | 3.25 | **29.74** | 8.34 | 19.23 | 9.62 |
| Stomach or other digestive disorders | 18.43 | **89.06** | 11.83 | 35.08 | 29.59 |
| Arthritis or rheumatism | 24.20 | **90.29** | 37.30 | 61.00 | 39.99 |
| Asthma | 0.61 | 2.81 | 1.21 | 74.21 | 5.71 |
| The bolded parts refer to the diseases that have been identified. | | | | |  |

| **Table S7. Longitudinal associations between LCA-derived multimorbidity classes and BADL disability** based on CHARLS 2011–2020 | | | |
| --- | --- | --- | --- |
|  | **Multimorbidity Patterns (OR [95% CI])** | | |
|  | **Visceral-Skeletal Diseases** | **Metabolic Diseases** | **Respiratory System Diseases** |
| **Model 1** | **1.22 (1.16–1.27)** | 1.01 (0.97–1.06) | **1.31 (1.21–1.42)** |
| **Time** | **1.70(1.68-1.73)** | | |
| **Model 2** | **1.09 (1.04–1.15)** | **0.91(0.86–0.95)** | **1.12 (1.03-1.22)** |
| **Time** | **1.69(1.66-1.71)** | | |
| **Model 3** | **1.07 (1.02–1.13)** | **0.82 (0.78-0.877)** | **1.012 (1.009–1.015)** |
| **Time** | **1.67(1.64-1.70)** | | |
| *P*<0.05 indicated in bold.  Model 1: adjusted for the score of other three multimorbidity patterns.  Model 2: in addition to Model 1, adjusted for age, gender, education level, marital status, and urban or rural residence.  Model 3: in addition to Model 2, adjusted for BMI. | | | |

| **Table S8**. Longitudinal associations between LCA-derived multimorbidity classes and IADL disability based on CHARLS 2011–2020 | | | |
| --- | --- | --- | --- |
|  | **Multimorbidity Patterns (OR [95% CI])** | | |
|  | **Visceral-Skeletal Diseases** | **Metabolic Diseases** | **Respiratory System Diseases** |
| **Model 1** | **1.65 (1.57–1.73)** | **1.23 (1.17–1.28)** | **2.07 (1.90–2.25)** |
| **Time** | **1.84(1.81-1.86)** | | |
| **Model 2** | **1.25 (1.19–1.31)** | **0.98(0.94–1.03)** | **1.48 (1.36-1.61)** |
| **Time** | **1.79(1.76-1.81)** | | |
| **Model 3** | **1.26 (1.19–1.32)** | **0.95 (0.90-0.99)** | **1.43 (1.31–1.56)** |
| **Time** | **1.74(1.72-1.77)** | | |
| *P*<0.05 indicated in bold.  Model 1: adjusted for the score of other three multimorbidity patterns.  Model 2: in addition to Model 1, adjusted for age, gender, education level, marital status, and urban or rural residence.  Model 3: in addition to Model 2, adjusted for BMI. | | | |

| **Table S9.** Longitudinal associations between multimorbidity patterns and BADL disability based on CHARLS 2011–2018(N=8129) | | | | |
| --- | --- | --- | --- | --- |
|  | **Multimorbidity Patterns (OR [95% CI])** | | | |
|  | **Visceral-Skeletal Diseases** | **Respiratory System Diseases** | **Neurodegenerative Diseases** | **Cardiometabolic Diseases** |
| **Model 1** |  |  |  |  |
| **Factor Score** |  |  |  |  |
| **T1** | 1.00 | 1.00 | 1.00 | 1.00 |
| **T2** | **1.69 (1.51-1.90)** | 1.06 (0.95-1.19) | 1.10 (0.98**-**1.23) | **1.42 (1.26-1.60)** |
| **T3** | **3.01 (2.67- 3.39)** | **1.16 (1.04 -1.29)** | **1.84 (1.64-2.06)** | **2.06 (1.82-2.33)** |
| ***P*_trend_** | **<0.001** | **0.016** | **<0.001** | **<0.001** |
| **Per Increase in Factor Score** | **2.97 (2.61-3.38)** | **1.69 (1.47-1.94)** | **5.28 (4.22-6.60)** | **2.13 (1.91- 2.38)** |
| **Model 2** |  |  |  |  |
| **Factor Score** |  |  |  |  |
| **T1** | 1.00 | 1.00 | 1.00 | 1.00 |
| **T2** | **1.75 (1.56-1.97)** | 1.04 (0.93**-**1.16) | **1.14 (1.01-1.27)** | **1.29 (1.14-1.45)** |
| **T3** | **3.16 (2.80-3.55)** | **1.11 (1.00-1.24)** | **1.83 (1.63-2.05)** | **1.88 (1.67-2.13)** |
| ***P*_trend_** | **<0.001** | 0.070 | **<0.001** | **<0.001** |
| **Per Increase in Factor Score** | **3.14 (2.77-3.57)** | **1.53 (1.34-1.75)** | **5.06 (4.05-6.32)** | **1.99 (1.78-2.22)** |
| **Model 3** |  |  |  |  |
| **Factor Score** |  |  |  |  |
| **T1** | 1.00 | 1.00 | 1.00 | 1.00 |
| **T2** | **1.81 (1.53-2.13)** | 1.09 (0.93**-**1.27) | 1.15 (0.98**-**1.34) | **1.08 (0.91-1.28)** |
| **T3** | **3.33 (2.81-3.94)** | **1.19 (1.03-1.38)** | **1.71(1.46-2.00)** | **1.48 (1.24-1.76)** |
| ***P*_trend_** | **<0.001** | 0.057 | **<0.001** | **<0.001** |
| **Per Increase in Factor Score** | **3.43 (2.88-4.08)** | **1.61 (1.34-1.92)** | **3.98 (2.94-5.39)** | **1.55 (1.32-1.83)** |
| *P*<0.05 indicated in bold.  Model 1: adjusted for the score of other three multimorbidity patterns.  Model 2: in addition to Model 1, adjusted for age, gender, education level, marital status, and urban or rural residence.  Model 3: in addition to Model 2, adjusted for BMI. | | | | |

| **Table S10.** Longitudinal association of multimorbidity patterns and IADL disability based on CHARLS 2011– 2018 (N=10011) | | | | |
| --- | --- | --- | --- | --- |
|  | **Multimorbidity Patterns (OR [95% CI])** | | | |
|  | **Visceral-Skeletal Diseases** | **Respiratory System Diseases** | **Neurodegenerative Diseases** | **Cardiometabolic Diseases** |
| **Model 1** |  |  |  |  |
| **Factor score** |  |  |  |  |
| **T1** | 1.00 | 1.00 | 1.00 | 1.00 |
| **T2** | **1.93 (1.73-2.15)** | **1.17 (1.05-1.31)** | **1.16 (1.04-1.30)** | **2.14 (1.90-2.40)** |
| **T3** | **3.22 (2.86-3.63)** | **1.56 (1.40-1.75)** | **1.99 (1.77- 2.24)** | **3.17 (2.80-3.59)** |
| ***P*_trend_** | **<0.001** | **<0.001** | **<0.001** | **<0.001** |
| **Per Increase in Factor Score** | **3.21 (2.80-3.68)** | **2.93 (2.52-3.40)** | **10.38 (8.07-13.35)** | **3.27 (2.91-3.67)** |
| **Model 2** |  |  |  |  |
| **Factor Score** |  |  |  |  |
| **T1** | 1.00 | 1.00 | 1.00 | 1.00 |
| **T2** | **1.79 (1.62-1.99)** | **1.11 (1.00-1.23)** | **1.27 (1.14-1.41)** | **1.67 (1.49-1.87)** |
| **T3** | **2.83 (2.53-3.16)** | **1.44 (1.30-1.60)** | **2.09 (1.87-2.34)** | **2.37 (2.11-2.66)** |
| ***P*_trend_** | **<0.001** | **<0.001** | **<0.001** | **<0.001** |
| **Per Increase in Factor Score** | **2.8 (2.48-3.17)** | **2.34 (2.04-2.68)** | **10.08 (7.98-12.72)** | **2.44 (2.19-2.71)** |
| **Model 3** |  |  |  |  |
| **Factor Score** |  |  |  |  |
| **T1** | 1.00 | 1.00 | 1.00 | 1.00 |
| **T2** | **1.84 (1.60-2.12)** | 1.15 (0.99**-**1.32) | **1.20 (1.04-1.39)** | **1.59 (1.36-1.87)** |
| **T3** | **2.86 (2.47-3.32)** | **1.50 (1.32-1.72)** | **1.94 (1.67-2.25)** | **2.24 (1.90-2.63)** |
| ***P*_trend_** | **<0.001** | **<0.001** | **<0.001** | **<0.001** |
| **Per Increase in Factor Score** | **2.78(2.37-3.27)** | **2.35 (1.96-2.78)** | **7.35 (5.41-9.99)** | **2.40 (2.06-2.79)** |
| *P*<0.05 indicated in bold.  Model 1: adjusted for the score of other three multimorbidity patterns.  Model 2: in addition to Model 1, adjusted for age, gender, education level, marital status, and urban or rural residence.  Model 3: in addition to Model 2, adjusted for BMI. | | | | |

| **Table S11.** Characteristics of the included and excluded populations for IADL during 2011-2020 | | | |
| --- | --- | --- | --- |
| **Characteristics** | **Exclusion (N=3967)** | **Inclusion(N=10051)** | ***P*** |
| **Age (years, mean±SD)** | 65.7±7.1 | 66.5±6.7 | **<0.001** |
| **Age group (years, %)** |  |  | 0.39 |
| **60–74** | 3388 (85.4%) | 8640 (86.0%) |  |
| **≥75** | 579 (14.6%) | 1411 (14.0%) |  |
| **Gender (%)** |  |  | **<0.001** |
| **Male** | 2344 (59.1%) | 4636 (46.1%) |  |
| **Female** | 1623 (40.9%) | 5415 (53.9%) |  |
| **Residential area (%)** |  |  | **<0.001** |
| **Urban** | 1900 (48.6%) | 3663 (36.8%) |  |
| **Rural** | 2013 (51.4%) | 6282 (63.2%) |  |
| **Marital status (%)** |  |  | **0.024** |
| **Married/Cohabiting** | 3279 (83.0%) | 8177 (81.4%) |  |
| **Not married** | 672 (17.0%) | 1874 (18.6%) |  |
| **Education level (%)** |  |  | **<0.001** |
| **Illiterate** | 888 (24.2%) | 3697 (36.8%) |  |
| **Elementary School or Below** | 1457 (39.7%) | 4291 (42.7%) |  |
| **Secondary School or Above** | 1321 (36.0%) | 2051 (20.4%) |  |
| **Smoking status (%)** |  |  | **<0.001** |
| **Never smoked** | 2040 (54.4%) | 5944 (62.0%) |  |
| **Former smoker** | 486 (12.9%) | 959 (10.0%) |  |
| **Current smoker** | 1227 (32.7%) | 2684 (28.0%) |  |
| **Drinking status (%)** |  |  | **<0.001** |
| **Do not drink** | 2186 (56.1%) | 6187 (61.6%) |  |
| **Drink, <1 time/month** | 351 (9.0%) | 739 (7.4%) |  |
| **Drink, ≥1 time/month** | 1360 (34.9%) | 3112 (31.0%) |  |
| **Sleep Hours (%)** |  |  | **0.30** |
| **<7** | 1734 (50.9%) | 4363 (52.1%) |  |
| **7–8** | 1366 (40.1%) | 3223 (38.5%) |  |
| **>8** | 310 (9.1%) | 782 (9.3%) |  |
| **BMI (kg/m^2^, mean±SD)** | 23.1±3.8 | 23.2±3.9 | **0.16** |
| **Physical activity (%)** |  |  |  |
| **High** | 418 (17.6%) | 1118 (23.5%) | **<0.001** |
| **Middle** | 1408 (59.2%) | 2523 (53.1%) |  |
| **Low** | 552 (23.2%) | 1110 (23.4%) |  |
| *P*<0.05 indicated in bold. | | | |

| **Table S12.** Longitudinal associations between multimorbidity patterns and BADL disability based on CHARLS 2011–2020 (N=10051) | | | | |
| --- | --- | --- | --- | --- |
|  | **Multimorbidity Patterns (OR [95% CI])** | | | |
|  | **Visceral-Skeletal Diseases** | **Respiratory System Diseases** | **Neurodegenerative Diseases** | **Cardiometabolic Diseases** |
| **Model 1** |  |  |  |  |
| **Factor Score** |  |  |  |  |
| **T1** | 1.00 | 1.00 | 1.00 | 1.00 |
| **T2** | **1.08 (1.01–1.15)** | **1.22 (1.15–1.29)** | 1.00 (0.94–1.06) | 0.98 (0.92–1.04) |
| **T3** | **1.29 (1.22–1.37)** | **1.21 (1.15–1.28)** | **1.67 (1.57–1.77)** | 1.00 (0.94–1.06**)** |
| ***P*_trend_** | **0.005** | **0.001** | **<0.001** | **<0.001** |
| **Per Increase in Factor Score** | **1.33 (1.25–1.41)** | **1.20 (1.12–1.29)** | **2.56 (2.29–2.85)** | **0.87 (0.83–0.92)** |
| **Model 2** |  |  |  |  |
| **Factor Score** |  |  |  |  |
| **T1** | 1.00 | 1.00 | 1.00 | 1.00 |
| **T2** | **1.09 (1.02–1.16)** | **1.20 (1.13–1.28)** | 1.02 (0.95–1.08) | **0.94 (0.89–0.99)** |
| **T3** | **1.33 (1.25–1.42)** | **1.19 (1.12–1.26)** | **1.68 (1.58–1.78)** | **0.98 (0.93–1.05)** |
| ***P*_trend_** | **0.004** | **0.005** | **<0.001** | **<0.001** |
| **Per Increase in Factor Score** | **1.39 (1.30–1.48)** | **1.15 (1.07–1.24)** | **2.52 (2.25–2.83)** | **0.87 (0.82–0.92)** |
| **Model 3** |  |  |  |  |
| **Factor Score** |  |  |  |  |
| **T1** | 1.00 | 1.00 | 1.00 | 1.00 |
| **T2** | **1.12 (1.04–1.20)** | **1.22 (1.14–1.31)** | 1.01 (0.94–1.08) | **0.86 (0.81–0.93)** |
| **T3** | **1.37 (1.28–1.46)** | **1.22 (1.14–1.30)** | **1.56 (1.46–1.67)** | **0.86 (0.80–0.93)** |
| ***P*_trend_** | **<0.001** | **<0.001** | **<0.001** | **<0.001** |
| **Per Increase in Factor Score** | **1.44 (1.34–1.55)** | **1.19 (1.10–1.29)** | **2.16 (1.90–2.46)** | **0.76 (0.71–0.81)** |
| Note: *P*<0.05 indicated in bold. The results were based on participants for whom fluctuating functional status was not considered.  Model 1: adjusted for the score of other three multimorbidity patterns.  Model 2: in addition to Model 1, adjusted for age, gender, education level, marital status, and urban or rural residence.  Model 3: in addition to Model 2, adjusted for BMI. | | | | |

| **Table S13.** Longitudinal association of multimorbidity patterns and IADL disability based on CHARLS 2011– 2020 (N=11461) | | | | |
| --- | --- | --- | --- | --- |
|  | **Multimorbidity Patterns (OR [95% CI])** | | | |
|  | **Visceral-Skeletal Diseases** | **Respiratory System Diseases** | **Neurodegenerative Diseases** | **Cardiometabolic Diseases** |
| **Model 1** |  |  |  |  |
| **Factor score** |  |  |  |  |
| **T1** | 1.00 | 1.00 | 1.00 | 1.00 |
| **T2** | **1.20 (1.14–1.27)** | **1.29 (1.22–1.36)** | 0.97 (0.92–1.03) | **1.15 (1.09–1.22)** |
| **T3** | **1.41 (1.33–1.50)** | **1.34 (1.27–1.42)** | **1.65 (1.55–1.75)** | **1.21 (1.14–1.28)** |
| ***P*_trend_** | **<0.001** | **<0.001** | **<0.001** | 0.959 |
| **Per Increase in Factor Score** | **1.45 (1.36–1.54)** | **1.48 (1.38–1.59)** | **2.84 (2.53–3.19)** | 1.01 (0.96–1.07**)** |
| **Model 2** |  |  |  |  |
| **Factor Score** |  |  |  |  |
| **T1** | 1.00 | 1.00 | 1.00 | 1.00 |
| **T2** | **1.17 (1.10–1.24)** | **1.23 (1.15–1.30)** | 1.01 (0.95–1.07**)** | **1.06 (1.01–1.13)** |
| **T3** | **1.38 (1.30–1.48)** | **1.27 (1.20–1.34)** | **1.63 (1.53–1.73)** | **1.15 (1.09–1.23)** |
| ***P*_trend_** | **<0.001** | **<0.001** | **<0.001** | 0.479 |
| **Per Increase in Factor Score** | **1.44 (1.36–1.54)** | **1.37(1.27–1.47)** | **2.73 (2.43–3.06)** | 1.00 (0.95–1.06**)** |
| **Model 3** |  |  |  |  |
| **Factor Score** |  |  |  |  |
| **T1** | 1.00 | 1.00 | 1.00 | 1.00 |
| **T2** | **1.20 (1.12–1.28)** | **1.25 (1.17–1.33)** | 1.01 (0.94–1.08) | 1.03 (0.97–1.10) |
| **T3** | **1.42 (1.33–1.52)** | **1.29 (1.21–1.37)** | **1.49 (1.39–1.59)** | **1.12 (1.04–1.20)** |
| ***P*_trend_** | **<0.001** | **<0.001** | **<0.001** | **0.003** |
| **Per Increase in Factor Score** | **1.49 (1.39–1.60)** | **1.37 (1.27–1.49)** | **2.17 (1.90–2.47)** | 0.98 (0.92–1.04) |
| Note: *P*<0.05 indicated in bold. The results were based on participants for whom fluctuating functional status was not considered.  Model 1: adjusted for the score of other three multimorbidity patterns.  Model 2: in addition to Model 1, adjusted for age, gender, education level, marital status, and urban or rural residence.  Model 3: in addition to Model 2, adjusted for BMI. | | | | |

| **Table S14.** Prevalence of chronic diseases in the older adults from 2011-2020 | | | | | | | | | | | | | | | |
| --- | --- | --- | --- | --- | --- | --- | --- | --- | --- | --- | --- | --- | --- | --- | --- |
| **Chronic Diseases** | **2011(N=7680)** | | | **2013(N=8733)** | | | **2015(N=9623)** | | | **2018(N=9634)** | | | **2020(N=9914)** | | |
|  | Yes | NO | prevalence | Yes | NO | prevalence | Yes | NO | prevalence | Yes | NO | prevalence | Yes | NO | prevalence |
| Hypertension | 3825 | 3855 | 49.8% | 5094 | 3639 | 58.3% | 5971 | 3652 | 62.0% | 6065 | 3569 | 62.9% | 4887 | 5027 | 49.3% |
| Dyslipidemia | 3422 | 4258 | 44.5% | 3898 | 4835 | 44.6% | 5607 | 4016 | 58.3% | 6030 | 3604 | 62.6% | 2838 | 7076 | 28.6% |
| Diabetes mellitus or elevated blood glucose | 3554 | 4126 | 46.3% | 3589 | 5144 | 41.1% | 4461 | 5162 | 46.4% | 4541 | 5093 | 47.1% | 1694 | 8220 | 17.1% |
| Chronic lung disorders | 1272 | 6408 | 16.6% | 1650 | 7083 | 18.9% | 1814 | 7809 | 18.9% | 2096 | 7538 | 21.8% | 1914 | 8000 | 19.3% |
| Liver disorders | 376 | 7304 | 4.9% | 549 | 8184 | 6.3% | 654 | 8969 | 6.8% | 925 | 8709 | 9.6% | 755 | 9159 | 7.6% |
| Heart disease | 1408 | 6272 | 18.3% | 1846 | 6887 | 21.1% | 2202 | 7421 | 22.9% | 2640 | 6994 | 27.4% | 2597 | 7317 | 26.2% |
| Stroke | 361 | 7319 | 4.7% | 496 | 8237 | 5.7% | 538 | 9085 | 5.6% | 1065 | 8569 | 11.1% | 997 | 8917 | 10.1% |
| Kidney disorders | 604 | 7076 | 7.9% | 860 | 7873 | 9.8% | 1089 | 8534 | 11.3% | 1408 | 8226 | 14.6% | 1169 | 8745 | 11.8% |
| Stomach or other digestive disorders | 1990 | 5690 | 25.9% | 2670 | 6063 | 30.6% | 3114 | 6509 | 32.4% | 3667 | 5967 | 38.1% | 3234 | 6680 | 32.6% |
| Emotional and psychiatric problems | 147 | 7533 | 1.9% | 153 | 8580 | 1.7% | 166 | 9457 | 1.7% | 272 | 9362 | 2.8% | 530 | 9384 | 5.3%% |
| Memory-related disorders | 295 | 7385 | 3.8% | 441 | 8292 | 5.0% | 481 | 9142 | 5.0% | 682 | 8952 | 7.1% | 729 | 9185 | 7.3% |
| Arthritis or rheumatism | 3312 | 4368 | 43.1% | 4140 | 4593 | 47.4% | 4709 | 4914 | 49.0% | 5130 | 4504 | 53.3% | 4336 | 5578 | 43.7% |
| Asthma | 550 | 7130 | 7.2% | 724 | 8009 | 8.3% | 781 | 8842 | 8.1% | 912 | 8722 | 9.5% | 811 | 9103 | 8.2% |

| **Table S15.** Characteristics of the 2011-2018 and 2020 (45586 observations) | | | |
| --- | --- | --- | --- |
| **Characteristics** | **2011-2018 (35670 observations)** | **2020(9916 observations)** | ***P*** |
| **Hypertension (%)** |  |  | **<0.001** |
| YES | 20955 (58.7%) | 4888 (49.3%) |  |
| NO | 14715 (41.3%) | 5028(50.7%) |  |
| **Diabetes mellitus or elevated blood glucose (%)** |  |  | **<0.001** |
| YES | 16145 (45.3%) | 1694 (17.8%) |  |
| NO | 19525 (54.7%) | 8222(82.9%) |  |
| **Dyslipidemia (%)** |  |  | **<0.001** |
| YES | 18957 (53.1%) | 2823 (28.6%) |  |
| NO | 16713 (46.9%) | 7078 (71.4%) |  |
| **Heart disease(%)** |  |  | **<0.001** |
| YES | 8096 (22.7%) | 2598 (26.2%) |  |
| NO | 27574 (77.3%) | 7318 (73.8%) |  |
| **BADL(%)** |  |  | **<0.001** |
| YES | 9171 (33.1%) | 6949(70.1%) |  |
| NO | 18522 (66.9%) | 2966(29.2%) |  |
| **IADL (%)** |  |  | **<0.001** |
| YES | 12959 (37.8%) | 6553(66.1%) |  |
| NO | 21374 (62.2%) | 3359(33.9%) |  |
| *P*<0.05 indicated in bold. | | | |
